# Body image disturbance and surgical decision making in egyptian post menopausal breast cancer patients

**DOI:** 10.1186/1477-7819-7-66

**Published:** 2009-08-13

**Authors:** Ashraf M Shoma, Madiha H Mohamed, Nashaat Nouman, Mahmoud Amin, Ibtihal M Ibrahim, Salwa S Tobar, Hanan E Gaffar, Warda F Aboelez, Salwa E Ali, Soheir G William

**Affiliations:** 1 Surgery Department, Mansoura University Hospital, Egypt; 2 Medical Surgical Department, Mansoura Faculty of Nursing, Egypt; 3 Psychiatric Department, Mansoura University Hospital, Egypt; 4 Medical Surgical Department, Alexandria Faculty of Nursing, Egypt

## Abstract

**Background:**

In most developing countries, as in Egypt; postmenopausal breast cancer cases are offered a radical form of surgery relying on their unawareness of the subsequent body image disturbance. This study aimed at evaluating the effect of breast cancer surgical choice; Breast Conservative Therapy (BCT) versus Modified Radical Mastectomy (MRM); on body image perception among Egyptian postmenopausal cases.

**Methods:**

One hundred postmenopausal women with breast cancer were divided into 2 groups, one group underwent BCT and the other underwent MRM. Pre- and post-operative assessments of body image distress were done using four scales; Breast Impact of Treatment Scale (BITS), Impact of Event Scale (IES), Situational Discomfort Scale (SDS), and Body Satisfaction Scale (BSS).

**Results:**

Preoperative assessment showed no statistical significant difference regarding cognitive, affective, behavioral and evaluative components of body image between both studied groups. While in postoperative assessment, women in MRM group showed higher levels of body image distress among cognitive, affective and behavioral aspects.

**Conclusion:**

Body image is an important factor for postmenopausal women with breast cancer in developing countries where that concept is widely ignored. We should not deprive those cases from their right of less mutilating option of treatment as BCT.

## Background

Breast cancer is the most common cancer in women in developed western countries [1] and is becoming even more significant in many developing countries [2]. In Egypt, breast cancer is the most common cancer among women, representing 18.9% of total cancer cases [3] with an age-adjusted rate of 49.6 per100 000 population [4].

Older women, who account for more than half of the new cases of breast cancer each year [5], are the fastest growing segment of the United States population [6]. Therefore, during the coming decades, older women will account for an increasing number of new cases and survivors [7]. At present, treatment for this growing diverse population is variable and represents evolving paradigms [8]. Decisions about optimal treatment patterns will ultimately depend on trial data about efficacy and woman's treatment preferences.

Several investigators dealing with early-stage breast cancer have two surgical options for treating local disease, breast conservative therapy (BCT) or mastectomy (MT) [9]. Because these treatments are equivalent with respect to survival, preferences for treatment may be important in quality-of-life (QOL) outcomes [10]. Preferences about maintaining body image are a key component in decision making for younger women [11].

Multiple studies have demonstrated that treatment for women with breast cancer differs substantially by patient age; with older women more likely to receive a more radical surgery [12]. This view is greatly adopted in many developing countries and the reasons for the difference are probably multifactorial including poorer performance status, less social support, difficulty with transportation, patient or family preference, negligence of QOL, lower life expectancy, and age bias [13]. In addition, few research studies have included older women, the lack of data may lead to less aggressive care.

Multiple studies had demonstrated that, women with BCT generally exhibit more positive body image [14]; they are less likely to become self-conscious about body presentation [15] or experience feelings of loss, and more likely to maintain feelings of physical attractiveness and femininity; compared with women who receive MT [16].

However, none of those studies focused on elderly women, leaving a large and growing segment of breast cancer survivors understudied with respect to body image preferences and postmenopausal QOL outcomes.

Therefore, our study was directed to compare the impact of the two surgical options, BCT versus MT, on body image disturbance among Egyptian postmenopausal breast cancer cases.

### Patients and Methods

We conducted a prospective randomized trial enrolled between February 2004 and December 2007. Briefly a sample of 100 post menopausal women with newly diagnosed stage I or II breast cancer was recruited from the surgical department of Mansoura University Hospital. Fifty cases underwent modified Radical Mastectomy (MRM). None of those cases had on table reconstruction. The other 50 patients had BCT. Women were excluded if they had chronic debilitating diseases e.g. heart disease or diabetes. Patients with chronic illness could face permanent changes in life-style, social stigma, dependency, self-management tasks, threats to dignity and diminished self-esteem, diagnostic uncertainties, disruption of normal life transitions and decreasing resources. These disease-associated stressors challenge patients' abilities to maintain emotional balance and a satisfactory self image and may disrupt future perspectives and proper evaluation [17]. Patients were also excluded if they had history of breast cancer or other cancers and if they had deformities or cosmetic problems especially in the face and other exposed areas. Changes in appearance or function may result in altered body image perception, and decrease satisfaction that may interfere with proper evaluation of any recent body image disturbances.

Sociodemographic data were collected and pre and post-operative assessments of body image distress were done using four scales; BITS, IES, SDS, and BSS. Ethical approval was obtained from Mansoura University Medical Ethical Committee. After a verbal and written consent was signed by the patient, data were collected through semi-structured psychiatric interviews and medical records. Sociodemographic data were collected including patient's age, level of education (illiterate women -those who can not read and write- received assistance from the psychiatrist in reading the scales with extreme effort not to interfere with the assessment), occupation, fear of recurrence, the degree of support provided by their partners and patients' believes about their illness.

Body image scales were introduced preoperatively. Another assessment by the same scales was done postoperatively after complete wound closure with no evidence of exudation, gaping or infection (usually the day after we remove the stitches, 10–15 days post operatively) to evaluate body image after actual changes caused by surgical intervention.

### Body image scales

There are four interrelated aspects of body image: cognitive, affective, behavioral, and evaluative components. Cognitive component is how accurately the person estimates his/her body size, either the entire body, or a particular body part. It is an interpretation of such external sensation as observing one's reflection or internal sensation. Affective body image is the emotional responses engendered by one's thoughts about the body. Behavioral component reflects actions about or toward the body. In another words, the activities engaged in or avoided depending on feeling toward one's own body. Evaluative component of body image is described as; person's rating of her/his body image [18].

Body image distress in breast cancer patients refers to subjective psychological stress that accompanies women's negative feelings, emotions, thoughts, and behaviors resultant from breast cancer and/or breast surgeries. We tried to use scales that cover these different components as much as possible. The following scales were used;

#### 1. Breast impact of treatment scale (BITS) [19]

Its item content was derived from prior breast cancer research assessing post-treatment concerns of women receiving breast surgery. It assesses the intrusive and avoidant response to the hypothesized traumatic event of surgical treatment of breast cancer (cognitive aspect). Intrusive response questions evaluate pervasive thoughts as "things I see or hear remind me that my body is different". Avoidant response questions measured limited cognitive experience, subjective awareness of emotions surrounding the event, as "I feel self conscious about letting my partner see my scar". It is a 15 item questionnaire, each item is weighed in 4 points scale (0 = not at all, 1 = rarely, 3 = sometimes, and 5 = often). Total score ranges from 0–75 with cut off point 26. This score indicates the severity of body image distress as following: 0–25 mild, 26–43 moderate, and 44+ severe ranges.

#### 2. Impact of Event Scale (IES) [20]

is a 15 item standardized self report questionnaire used to measure current subjective stress related to a specific event (affective aspect) e.g. "I had waves of strong feelings about it and I knew that a lot of unresolved feelings were still there, but I kept them under wraps". Women rate the frequency of these 15 feelings or events during past seven days using a 4 points scale (not at all = 0, rarely = 1, sometimes = 3, and often = 5). Total score ranges from 0–75 with cut off point 26. This score indicates the severity of body image distress as following: 0–25 mild, 26–43 moderate, and 44+ severe ranges.

#### 3. Situational Discomfort Scale (SDS) [21]

consists of five items based on retrospective psychosocial research on distressing situations following breast cancer surgeries (behavioral aspect). Participants rated their current level of distress across five situations (looking at your chest in the mirror when you are unclothed, undressed in front of other women, undressed in front of your partner, letting other women see the surgical site, and letting partner see the surgical site). Using a 5-point scale (1 = not at all distressed, 2 = a little distressed, 3 = somewhat distressed, 4 = moderately distressed, 5 = extremely distressed) the five situational discomfort items were summated to obtain a total distress score (range 5–25) and higher scores represent greater distress.

#### 4. Body Satisfaction Scale (BSS) [22]

is an abbreviated form consisting of 10 items. It measures the external body satisfaction following surgical procedures (evaluative aspect). Factor analysis has yielded two factors: The first one deals with Satisfaction with Appearance and the second factor deals with Weight or Body Correlates of Weight. In addition, a single item assessed satisfaction with overall appearance. The items of this scale were rated on a six points satisfaction/dissatisfaction scale (1 = extremely satisfied, 2 = moderately satisfied, 3 = satisfied, 4 = dissatisfied, 5 = moderately dissatisfied, 6 = extremely dissatisfied) with a higher score indicating greater body dissatisfaction.

Some statements that show differences between the four scales are listed in table 1.

**Table 1:** Examples of the statements of each scale used

**Breast impact of treatment scale (BITS)**	**Impact of Event Scale (IES)**	**Situational Discomfort Scale (SDS)**	**Body Satisfaction Scale (BSS)**
***Intrusive response questions:*** - Things I see or hear remind me that my body is different. - How my body has changed pops into my mind. ***Avoidant response questions: ***measured limited cognitive experience, subjective awareness of emotions surrounding the event, as "I feel self conscious about letting my partner see my scar", denial surrounding the event as "I avoid looking at and touching my scar"	- I had waves of strong feelings about it and I knew that a lot of unresolved feelings were still there, but I kept them under wraps. - I had dreams about it. - I felt as if it hadn't happened or wasn't real. - I was aware that I still had a lot of feelings about it, but I didn't deal with them.	- looking at your chest in the mirror when you are unclothed. - Undressed in front of other women. - Undressed in front of your partner. - Letting other women see the surgical site. - Letting partner see the surgical site.	Pick the description which currently prescribes how you regard your body: Head. The size of your breast. Hips. The shape of your breast. Genitals. Hair. Abdomen. Buttocks. Complexion. Weight. General. Appearance.

### Statistical analysis

Collected data were coded and then analyzed using the statistical package for the social sciences (SPSS) for windows (version 10.0) to test the statistical significant difference between groups. The description of data was done in form of mean ± standard deviation (SD) and frequency & proportion for qualitative data. Chi-square test was conducted to investigate qualitative data. Student t-test was conducted to investigate quantitative data between the two groups. Significant level of P is ≤ 0.05 at confidence interval 95%.

## Results

The patients' age ranged from 43 to 82 years old with a mean of 54.28 years and SD of ± 8.84 years. Patients in BCT group were slightly older than patients in mastectomy group. The mastectomy group contained a larger proportion of illiterate women (70%). In BCT group, women were more likely to report fear of recurrence than women in mastectomy group and slightly exhibit more supportive relationships with their partners. There were no differences between the two groups regarding percentage of working women or acceptance of reality about their illness (Table 2).

**Table 2:** Demographic data of the studied patients

**Sociodemographic characteristics**	**BCT**		**MRM**	
	
	**N = 50**	**%**	**N = 50**	**%**
**Age**				
43–50	7	14	15	30
51–60	17	34	18	36
61–70	17	34	15	30
71–82	9	18	2	4

**Level of education**				
Illiterate	28	56	35	70
Literate	22	44	15	30

**Occupation**				
House wife	38	76	39	78
Working	12	24	11	22

**Relation with partner**				
single	8	16	3	6
not supportive	6	12	15	30
supportive	16	32	16	32
very supportive.	20	40	16	32

**Fear from recurrence**				
Yes	41	82	21	42
No	9	18	29	58

**Believes of illness**				
Not accepted	14	28	14	28
Accepted	36	72	36	72

**BCT**: Breast conserving therapy

**MRM**: Modified radical mastectomy

**Figure 1:**
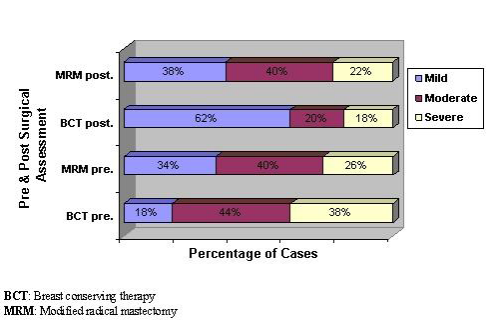
**Assessment of cognitive impact of body image distress during pre and postoperative period for both studied groups**.

The Chi square measurement of cognitive impact of body image distress preoperatively, showed no significant statistical difference (X^2 ^= 3.682, p = 0.159) between both studied groups. However, postoperatively it showed significant statistical difference (X^2 ^= 6.413, p = 0.040) where more than half of the patients (62%) in BCT group showed mild degree of distress while most of the patients in MRM group showed moderate (40%) and severe (22%) degree of distress (Figure 1).

**Figure 2:**
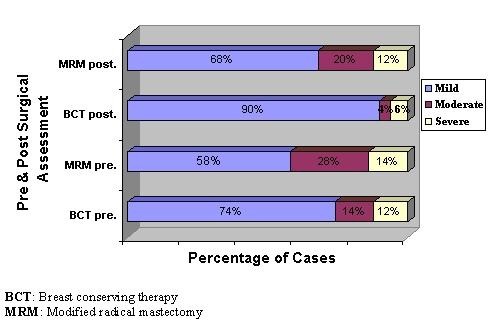
**Assessment of affective impact of body image distress during pre and postoperative period for both studied groups**.

Regarding measurement of the affective impact preoperatively, the Chi square showed no significant statistical difference (X^2 ^= 3.380, p = 0.185) between both studied groups. Postoperatively it showed significant statistical difference (X^2 ^= 7.865, p = 0.020) between both studied groups where only 10% of the patients in the BCT showed moderate (4%) and severe (6%) degree of affective distress while 32% of the patients in the MRM group showed moderate (20%) and severe (12%) degree of distress (Figure 2).

**Figure 3:**
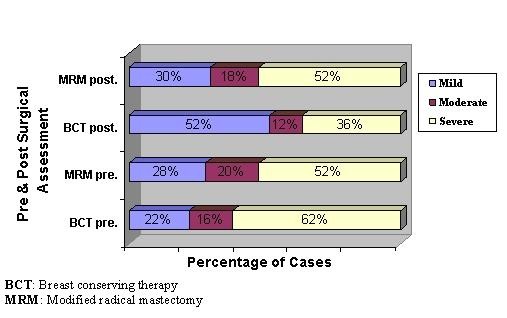
**Assessment of behavioral impact of body image distress during pre and postoperative period for both studied groups**.

Similarly the Chi square measurement of the behavioral impact preoperatively showed no significant statistical difference (X^2 ^= 1.021, p = 0.600) between both studied groups. Postoperatively, it showed significant statistical difference (X^2 ^= 6.006, p = 0.05) between both studied groups where more than half of the patients (52%) in BCT group showed mild degree of distress while more than half of the patients (52%) in MRM group showed severe degree of distress (Figure 3).

**Figure 4:**
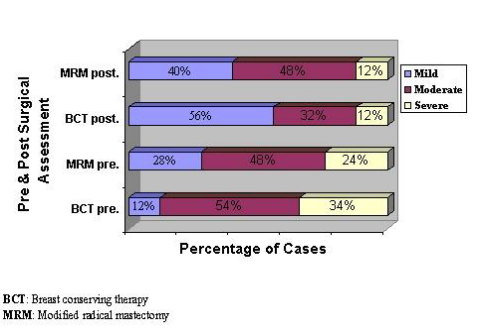
**Assessment of evaluative impact of body image distress during postoperative period for both studied groups**.

On the other hand, the Chi square measurement of the evaluative impact preoperatively, showed no significant statistical difference between both studied groups either pre (X^2 ^= 4.239, p = 0.120) or postoperatively (X^2 ^= 2.933, p = 0.231) (Figure 4).

The mean scores of impact of distress of the four components of body image distress both pre and postoperatively are shown in table 3. In BCT group there were significant statistical differences between the pre and post-operative mean scores of cognitive, affective, and behavioral impacts as well as in the total mean score (t = 0.52, p = < 0.001). On the other hand, the MRM group showed only a significant difference between pre and post-operative mean score of the affective impact (t = 0.52, p = < 0.001).

**Table 3:** Comparison between BCT and MRM groups as regards level of body image distress during pre and postoperative period.

	**BCT**				**MRM**			
						
	**Pre-operative Mean **± **SD**	**Post-operative Mean **± **SD**	**t-test**	**p**	**Pre-operative Mean **± **SD**	**Post-operative Mean **± **SD**	**t-test**	**P**
**Cognitive impact**	48.420 ± 14.331	33.08 ± 18.560	5.87	< 0.001*	44.62 ± 16.261	40.7 ± 18.202	1.45	0.15

**Affective impact**	49.840 ± 12.819	33.08 ± 5.566	10.85	< 0.001*	45.94 ± 13.287	38.9 ± 4.377	4.89	< 0.001*

**Behavioral impact**	19.18 ± 6.862	13.66 ± 8.250	4.69	< 0.001*	17.52 ± 7.791	17.1 ± 7.475	0.35	0.73

**Evaluative impact**	32.220 ± 9.740	33.82 ± 12.166	1.22	0.225	33.66 ± 14.394	35.5 ± 14.185	0.82	0.41

**Total mean**	37.41 ± 10.93	28.41 ± 11.13	5.2	< 0.001*	35.43 ± 12.93	33.05 ± 11.05	1.27	0.21

**BCT**: Breast conserving therapy

**MRM**: Modified radical mastectomy

(*) statistically highly significant (P < 0.01)

**SD**: Standard deviation

## Discussion

Although breast cancer continues to be the most common malignant tumor among women, it is a highly treatable disease [23]. MT (radical MT or MRM) was the treatment of choice for breast cancer regardless of the patient's age. At present, it is well accepted that BCT is equivalent to MT in terms of survival for early-stage breast cancer [24-27]. However a number of factors can influence treatment choice, including patient preferences, tumor and patient physical characteristics, and associated medical factors. Patient preference is often the most difficult aspect of eligibility determination [28]. Therefore, BCT is widely favored because, based on the emotional attachment to this organ [29], it is seen as less mutilating than MT [30]. Early comparisons of BCT with MT did not demonstrate major psychological advantages. However, more recently, cosmetic results [31] and patient satisfaction [32] following wide local excision were reported, and showed that the psychological outcome was better among patients with better cosmoses [33].

Curran and associates [34] reported that women in the BCT group had better body image and were more satisfied with treatment (p = 0.001) than those in the MT group. Similarly Hopwood et al., 2007 found a clinically significant increase in body image problems for women undergoing MT compared with BCT [35]. Rowland et al., 2000 also found that women who had BCT reported statistically significantly fewer problems with their body image than women who had MT [36]. Many other researches came to the same conclusion [37-40].

Previous researches concerning body image in patients treated for breast cancer primarily included younger and middle-aged women (mean age < 55 years) [41-44] and rarely included elderly women (mean age > 65 years) [45]. It is not certain whether findings from these studies of younger and middle-aged women can be accurately extrapolated to an elderly population [46]. Another limitation of prior researches is that most studies were quantitative in nature and few qualitative studies had specifically studied postmenopausal women's experience of breast cancer treatment [47] or those from developing countries.

In Egypt, like many other developing countries [48], most of the people think that a postmenopausal woman had finished her maternal role and it won't make a difference for her to have her breast removed. Traditions and taboos in these communities ignore the impact of removing an organ that represents a part of her identity and self regardless of her age. In our study, this was evaluated by using four scales in an attempt to cover the four aspects of the body image.

Comparing both groups on dimensions of body image distress revealed that in preoperative assessment, there was no statistical significant difference as regarding cognitive, affective, behavioral and evaluative impacts. As for cognitive impact; the majority of women in both BCT and MRM groups had negative thoughts regarding their experience with breast cancer. While the affective impact assessment for both studied groups expressed strong negative feelings. The behavioral impact assessment showed that the majority of both studied groups stated that, they become severely distressed on undressing in front of their partners. These results are in agreement with Perry et al (2007) who stated that, as many as 80% of patients with breast cancer report significant distress after diagnosis and during the initial treatment period, and consider feeling of shock, numbness, and anxiety about the future treatment and prognosis are normal to receive diagnosis of cancer [49].

In postoperative assessment, postmenopausal women in MRM group showed greater level of distress as regarding cognitive, affective and behavioral components. As for self evaluative impact, there was no significant statistical difference. The greater level of body image distress was in the behavioral component where women in both groups rated their level of distress across five situations in which they had either to see their scar or let others see it. As MRM is more disfiguring than BCT, more than half of the women who had MRM had severe degree of distress as regarding behavioral component. These results indicate that postmenopausal women receiving MRM showed a significantly less favorable body image compared with those treated with BCT. So it appears that it is not the cancer that causes of body change distress, but it is the treatment. Also it appears that postmenopausal cases exhibit body image distress as premenopausal ones, so age itself should not be a contra-indication for conservative surgery.

On the contrary, Pozo et al., 1992 found no difference between BCT and MRM as regarding body image. This was explained by assuming the greatest concern for most patients is "they have cancer and they are trying to survive it" [50]. Poulsen and colleagues [51] also reported no significant differences between the 2 types of surgery on measures of body image. But this study differs from ours as they restrict its inclusion criteria to age ≤ 69 years, so the above results does not express the effect on postmenopausal women only as the study included younger women also with exclusion of large number of the post menopausal women. Also they used Linear Analogue Self-Assessment Scale (LASA) where six quality-of-life domains were assessed which did not give them the opportunity to examine body change from its different aspects.

It should be noted that most of the published studies that showed no protection from psychological dysfunction with BCT could have been due to "worry about a cancer recurrence" because only a small portion of the breast is excised. However, our study showed although postmenopausal women in BCT group showed less body image distress, they showed more fear of recurrence (82%) in comparison to MRM group (42%). "Fear of recurrence" has been at the heart of the controversy between surgeons favoring MT versus those advocating BCT. In fact, in the review of Kiebert and associates [52], six out of the eight studies which investigated fear of recurrence and death showed no difference between the two treatment strategies and the remaining two trials found more fear of recurrence after MRM than after BCT. The review of Schover [53] included six studies which produced conflicting results with respect to fear of recurrence; two showed no difference, one favored MRM and three favored BCT.

The importance of the significant other's support in illness recovery is well-documented [54]. Previous findings suggested that psychosocial interventions that improve both the person with cancer and the partner's social and emotional well-being may have positive effects on QOL [55,56]. The degree of the partner's emotional involvement and understanding of the woman's experience is directly associated with psychological adjustment [57]. In our study women in the BCT group showed more support from their partners than women in MRM, this can be of a special concern in Egypt where human relations and familial bonds are still so strong.

Postmenopausal women in BCT group were more educated (44%) in comparison to 70% illiterates in MRM group. Education can affect the patient decision about treatment a consequently affect body image or it may directly affect the cognitive appraisal of their new stressful situation. Roland et al (2000) emphasize that women undergoing MT with breast reconstruction and BCT are more likely to be highly educated [45]. On the other hand illiterates may leave the decision of the kind of operative intervention to significant others in their lives, unaware about the later psychological impact. Women's level of education is considered a predictor for stress in women with breast cancer, as less formal education is associated with poorer psychological adjustment, including attempts to cope with the stress of breast cancer by avoiding emotions, thoughts, or information related to the disease [58].

In some patients, denial may prevent them from making realistic plans for treatment. Peck suggested that women' use of denial, as a defense mechanism in the immediate post-operative period, may help them to come to terms with their new body image. However, over time, denial is difficult to sustain and patients may be forced to face progressively the reality of breast loss, which may result in loss of self-image satisfaction [59]. In our study we could not relate denial of illness to more body image distress as the percentage of denial in both groups was similar.

Our main concern was to explore the taboo of breast cancer surgical treatment in developing countries as it continues to have a deep impact on both patient's survival and body image disturbances. Physicians working in a limited resources environment may be forced to make decisions contrary to their best medical knowledge because diagnostic and/or treatment resources are lacking. For instance, lack of radiotherapy facilities prevents the use of BCT [60]. The patients' level of education, fear of recurrence, partner support and other factors may affect the surgical decision making. Our study demonstrates that prior assumption about body image not being important to post menopausal women in developing countries, is not true. Subsequently, those patients should be offered BCT as often as it is offered to younger women.

## Conclusion

Body image is an important aspect of the human psyche, and is not an issue reserved for developed countries only. It is time to change the concept of relying on age or menopausal status in surgical decision making. Postmenopausal breast cancer cases in developing countries have their concerns about body image and they have the right to be offered a less mutilating form of breast surgery once indicated. Oncology professionals caring for postmenopausal women with breast cancer need to be aware of a woman's preference about appearance and body image at the time of treatment decision making to assist in her choice of treatment and long-term adjustment.

## Competing interests

The authors declare that they have no competing interests.

## Authors' contributions

AMS was involved in the design of the study and writing of the manuscript. MHH, IMI, NN, MA, SST, and HEG assembled the data and performed the statistical analysis. WFA, SEA and SGW designed the study and assembled the data. All authors read and approved the final manuscript.

## References

[B1] Althuis MD, Dozier JM, Anderson WF, Devesa SS, Brinton LA (2005). Global trends in breast cancer incidence and mortality 1973–1997. Int J Epidemiol.

[B2] Yang L, Parkin DM, Ferlay J, Li L, Chen Y (2005). Estimates of cancer incidence in China for 2000 and projections for 2005. Cancer Epidemiol Biomarkers Prev.

[B3] Elatar I Cancer registration, NCI Egypt 2001. Cairo, Egypt, National Cancer Institute 2002. http://www.nci.edu.eg/Journal/nci2001%20.pdf.

[B4] Ibrahim AS (2002). Cancer profile in Gharbiah, Egypt. Methodology and Results. Cairo, Ministry of Health and Population Egypt and Middle East Cancer Consortium.

[B5] National Cancer Institute (2001). DCCPS Surveillance Research Program: Cancer Statistics Branch, released April. Surveillance, Epidemiology, and End Results (SEER) Program Public-Use Data (1973–1998).

[B6] Soldo BJ, Agree EM (1988). America's Elderly. Washington, DC, Population Reference Bureau.

[B7] Lash TL, Silliman RA (1998). Prevalence of cancer. J Natl Cancer Inst.

[B8] Hughes K, Schnaper L, Berry D, Cirrincione C, McCormick B, LU J, Smith T, Smith B, Shank B, Shapero C (2001). Comparison of lumpectomy plus tamoxifen with and without radiotherapy (RT) in women 70 years of age and older who have clinical stage I, estrogen receptor positive (ER+) breast carcinoma. Proc Am Soc Clin Oncol (ASCO).

[B9] Fisher B, Redmond C, Poisson R, Margolese R, Wolmark N, Wickerham L, Fisher E, Deutsch M, Caplan R, Pilch Y (1989). Eight-year Results of a randomized clinical trial comparing total mastectomy and segmental mastectomy with or without radiation in the treatment of breast cancer. N Engl J Med.

[B10] Mandelblatt JS, Edge SB, Meropol NJ, Senie R, Tsangaris T, Grey L, Peterson BM, Hwang YT, Kerner J, Weeks J (2003). Predictors of long-term outcomes in older breast cancer survivors: Perceptions versus patterns of care. J Clin Oncol.

[B11] Stokes R, Frederick-Recascion C (2003). Women's perceived body image: Relations with personal happiness. J Women Aging.

[B12] DeMichele A, Putt M, Zhang Y, Glick JH, Norman S (2003). Older age predicts a decline in adjuvant chemotherapy recommendations for patients with breast carcinoma: Evidence from a tertiary care cohort of chemotherapy-eligible patients. Cancer.

[B13] Muss HB (1995). Chemotherapy of breast cancer in the older patient. Semin Oncol.

[B14] Moyer A (1997). Psychsocial outcomes of Breast-conserving surgery: A meta-analytic review. Health Psychol.

[B15] Ganz PA, Schag CAC, Lee JJ, Polinsky ML, Tan SJ (1992). Breast conservation versus mastectomy: Is there a difference in psychological adjustment or quality of life in the year after surgery?. Cancer.

[B16] Deadman JM, Dewey MJ, Owens RG, Leinster SJ, Slade PD (1989). Threat and lost of breast cancer. Psychol Med.

[B17] Devins DM, Binik YM, Zeidner M, Endler NS (1996). Facilitating coping with chronic physical illness. Handbook of coping: Theory, research, application.

[B18] Banfield SS, McCabe MP (2002). An evaluation of the construct of body image. Adolescence.

[B19] Frierson GM, Thiel DL, Andersen BL (2006). Body Change Stress for Women with Breast Cancer: The Breast-Impact of Treatment Scale. Ann Behav Med.

[B20] Horowitz M, Wilner N, Alvarez W (1979). Impact of Events Scale: A measure of subjective stress. Psychosomatic Medicine.

[B21] Beckmann J, Johansen L, Blichert-Toft M (1983). Psychological reactions in younger women operated on for breast cancer. Danish Medical Bulletin.

[B22] Andersen BL, LeGrand J (1991). Body image for women: Conceptualization, assessment, and a test of its importance to sexual dysfunction and medical illness. The Journal of Sex Research.

[B23] Jemal A, Murray T, Samuels A, Ghafoor A, Ward E, Thun MJ (2003). Cancer statistics. CA Cancer J Clin.

[B24] Arriagada R, Lê MG, Rochard F, Contesso G (1996). Conservative treatment versus mastectomy in early breast cancer: patterns of failure with 15 years of follow-up data. Institut Gustave-Roussy Breast Cancer Group. J Clin Oncol.

[B25] Fisher B, Jeong JH, Anderson S, Bryant L, Fisher E, Wolmark L (2002). Twenty-five-year follow-up of a randomized trial comparing radical mastectomy, total mastectomy, and total mastectomy followed by irradiation. N Engl J Med.

[B26] Poggi MM, Danforth DN, Sciuto LC, Smith SL, Steinberg SM, Liewehr DJ, Menard C, Lippman ME, Lichter AS, Altemus RM (2003). Eighteen-year Results in the treatment of early breast carcinoma with mastectomy versus breast conservation therapy: the National Cancer Institute Randomized Trial. Cancer.

[B27] van Dongen JA, Voogd AC, Fentiman IS, Legrand C, Sylvester RJ, Tong D, Schueren E van der, Helle PA, van Zijl K, Bartelink H (2000). Long-term Results of a randomized trial comparing breast-conserving therapy with mastectomy: European Organization for Research and Treatment of Cancer 10801 trial. J Natl Cancer Inst.

[B28] NIH Consensus Conference (1991). Treatment of early stage breast cancer. JAMA.

[B29] Bartelink H, van Dam F, van Dongen J (1985). Psychological effects of breast conserving therapy in comparison with radical mastectomy. Int J Radiat Oncol Biol Phys.

[B30] Lasry JC, Margolese RG, Poisson R, Shibata H, Fleischer D, Lafleur D, Legault S, Taillefer Sl (1987). Depression and body image following mastectomy and lumpectomy. J Chronic Dis.

[B31] Al-Ghazal SK, Blamey RW, Stewart J, Morgan DAL (1999). The cosmetic outcome in early breast cancer treated with breast conservation. Eur J Surg Oncol.

[B32] Al-Ghazal SK, Fallowfield L, Blamey RW (1999). Patient evaluation of cosmetic outcome after conserving surgery for treatment of primary breast cancer. Eur J Surg Oncol.

[B33] Al-Ghazal SK, Fallowfield L, Blamey RW (1999). Does cosmetic outcome from treatment of primary breast cancer influence psychosocial morbidity?. Eur J Surg Oncol.

[B34] Curran D, van Dongen JP, Aaronson NK, Kiebert G, Fentiman IS, Mignolet F, Bartelink H (1998). Quality of life of early-stage breast cancer patients treated with radical mastectomy or breast-conserving procedures: Results of EORTC Trial 10801. The European Organization for Research and Treatment of Cancer (EORTC), Breast Cancer Co-operative Group (BCCG). Eur J Cancer.

[B35] Hopwood P, Haviland J, Mills J, Sumo G, M Bliss J, START Trial Management Group (2007). The impact of age and clinical factors on quality of life in early Breast cancer: An analysis of 2208 women recruited to the UK START Trial (Standardization of Breast Radiotherapy Trial). The Breast.

[B36] Rowland JH, Desmond KA, Meyerowitz BE, Belin TR, Wyatt GE, Ganz PA (2000). Role of Breast Reconstructive Surgery in Physical and Emotional Outcomes Among Breast Cancer Survivors. Journal of the National Cancer Institute.

[B37] Yurek D, Farrar W, Andersen BL (2000). Comparing Surgical Groups and Determining Individual Differences in Postoperative Sexuality and Body Change Stress. J Consult Clin Psychol.

[B38] Kenny P, King LM, Shiell A, Seymour J, Hall J, Langlands A, Boyages J (2000). Early stage breast cancer: costs and quality of life one year after treatment by mastectomy or conservative surgery and radiation therapy. The Breast.

[B39] De Haes JCJM, Curran D, Aaronson NK, Fentiman IS (2003). Quality of life in breast cancer patients aged over 70 years, participating in the EORTC 10850 randomised clinical trial. European Journal of Cancer.

[B40] Figueiredo MI, Cullen J, Hwang Y, Rowland JH, Mandelblatt JS (2004). Breast Cancer Treatment in Older Women: Does Getting What You Want Improve Your Long-Term Body Image and Mental Health?. Journal of Clinical Oncology.

[B41] Badger T, Segrin C, Meek P, Lopez AM, Bonham E (2004). A case study of telephone interpersonal counseling for women with breast cancer and their partners. Oncology Nursing Forum.

[B42] Fortner BV, Stepanski EJ, Wang SC, Kasprowicz S, Durrence HH (2002). Sleep and quality of life in breast cancer patients. Journal of Pain Symptom Management.

[B43] Dibble SL, Israel J, Nussey B, Casey K, Luce J (2003). Delayed chemotherapy-induced nausea in women treated for breast cancer. Oncology Nursing Forum.

[B44] Beck S, Dudley WN, Barsvick A (2005). Pain, sleep disturbance, and fatigue in patients with cancer: Using a mediation model to test a symptom cluster. Oncology Nursing Forum.

[B45] Rao A, Cohen HJ (2004). Symptom management in the elderly cancer patient: Fatigue, pain, and depression. Journal of the National Cancer Institute Monographs.

[B46] Yanick R, Wesley MN, Ries LA, Havlik RJ, Edwards BK, Yates JW (2001). Effect of age and co-morbidity in postmenopausal breast cancer patients aged 55 year and older. JAMA.

[B47] Thewes B, Butow P, Pendlebury S (2004). The psychosocial needs of breast cancer survivors: a qualitative study of the shared and unique needs of younger versus older survivors. Psycho-Oncology.

[B48] Al-Moundhri M, Al-Bahrani B, Pervez I, Ganguly SS, Nirmala V, Al-Madhani A, Al-Mawaly K, Grant C (2004). The outcome of treatment of breast cancer in a developing country Oman. The Breast.

[B49] Perry S, Kowalski T, Chang C (2007). Quality of life assessment in women with breast cancer: benefits, acceptability and utilization. Health Qual Life Outcomes.

[B50] Pozo C, Carver CS, Noriega V, Harris SD, Robinson DS, Ketcham AS, Legaspi A, Moffat FL, Clark KC (1992). Effects of Mastectomy versus Lumpectomy on Emotional Adjustment to Breast Cancer: A Prospective Study of the First Year Postsurgery. J Clin Oncol.

[B51] Poulsen B, Graversen HP, Beckmann J, Blichert-Toft M (1997). A comparative study of post-operative psychosocial function in women with primary operable breast cancer randomized to breast conservation therapy or mastectomy. Eur J Surg Oncol.

[B52] Kiebert GM, de Haes JCJM, Velde CJH van de (1991). The impact of breast-conserving treatment and mastectomy on the quality of life of early breast cancer patients: a review. J Clin Oncol.

[B53] Schover LR (1991). The impact of breast cancer on sexuality, body image, and intimate relationships. A Cancer J Clin.

[B54] Han WT, Collie K, Koopman C, Azarow J, Classen C, Morrow GR, Michel B, Brennan-O'Neill E, Spiegel D (2005). Breast cancer and problems with medical interactions: Relationships with traumatic stress, emotional self-efficacy, and social support. Psycho-Oncology.

[B55] Segrin C, Badger TA, Sieger A, Meek P, Lopez AM (2006). Interpersonal well-being and mental health among male partners of women with breast cancer. Issues in Mental Health Nursing.

[B56] Segrin C, Badger TA, Meek P, Lopez AM, Bonham E, Sieger A (2005). Dyadic interdependence on affect and quality of life trajectories among women with breast cancer and their partners. Journal of Social and Personal Relationships.

[B57] Wimberly SR, Carver CS, Laurenceau J, Harris SD, Antoni MH (2005). Perceived partner reactions to diagnosis and treatment of breast cancer: Impact on psychosocial and psychosexual adjustment. J Consult Clin Psychol.

[B58] Fobair P, Stewart SL, Chang S, D'onofrio C, Banks PJ, Bloom JR (2006). body image and sexual problems in younger women withy breast cancer. Psychooncology.

[B59] Peck A (1974). Psychological effects of radical mastectomy immediately after biopsy. JAMA, J Am Med Assoc.

[B60] Bese NS, Kiel K, El-Gueddari BE-K, Campbell OB, Awuah B, Vikram B, International Atomic Energy Agency (2006). Radiotherapy for breast cancer in countries with limited resources: Program implementation and evidence-based recommendations. Breast J.

